# Prenatal Maternal Stress Predicts Childhood Asthma in Girls: Project Ice Storm

**DOI:** 10.1155/2014/201717

**Published:** 2014-05-08

**Authors:** Anne-Marie Turcotte-Tremblay, Robert Lim, David P. Laplante, Lester Kobzik, Alain Brunet, Suzanne King

**Affiliations:** ^1^University of Montreal Hospital Research Centre, Montreal, QC, Canada H2X 0A9; ^2^University of Montreal, Montreal, QC, Canada H3N 1X9; ^3^Douglas Hospital Research Center, Montreal, QC, Canada H4H 1R3; ^4^Harvard School of Public Health, Boston, MA 02115, USA; ^5^Boston Children's Hospital, Boston, MA 02115, USA; ^6^Brigham and Women's Hospital, Boston, MA 02115, USA; ^7^McGill University, Montreal, QC, Canada H3A 1A1

## Abstract

Little is known about how prenatal maternal stress (PNMS) influences risks of asthma in humans. In this small study, we sought to determine whether disaster-related PNMS would predict asthma risk in children. In June 1998, we assessed severity of objective hardship and subjective distress in women pregnant during the January 1998 Quebec Ice Storm. Lifetime asthma symptoms, diagnoses, and corticosteroid utilization were assessed when the children were 12 years old (*N* = 68). No effects of objective hardship or timing of the exposure were found. However, we found that, in girls only, higher levels of prenatal maternal subjective distress predicted greater lifetime risk of wheezing (OR = 1.11; 90% CI = 1.01–1.23), doctor-diagnosed asthma (OR = 1.09; 90% CI = 1.00–1.19), and lifetime utilization of corticosteroids (OR = 1.12; 90% CI = 1.01–1.25). Other perinatal and current maternal life events were also associated with asthma outcomes. Findings suggest that stress during pregnancy opens a window for fetal programming of immune functioning. A sex-based approach may be useful to examine how prenatal and postnatal environments combine to program the immune system. This small study needs to be replicated with a larger, more representative sample.

## 1. Introduction

Currently affecting 235 million people worldwide, asthma is the most common chronic disease among children [1]. Neither its causes nor the reasons for its increasing prevalence are completely understood. Researchers are increasingly turning to perinatal factors to explain variance in childhood immune functions [2–5] and risk for asthma [6–12]. Self-reported prenatal maternal anxiety was found to explain 9.3% of the variance in risk for respiratory illnesses [13] and to predict asthma in offspring [14]. Moreover, prenatal community violence, a psychosocial stressor, was found to be associated with wheezing at the age of two years [15].

The biological mechanisms by which prenatal maternal stress (PNMS) affects the fetal immune system are unclear and may differ based on the type, duration, frequency, and severity of the stressor and the sex of the child [16]. Stressful events activate the hypothalamic-pituitary-adrenal (HPA) axis and trigger the secretion of glucocorticoids (GC) that may reach the fetus by transplacental passage, permanently altering fetal HPA axis and immune functions [17].

Strong evidence supports the notion that PNMS has different programming effects on male and female animals [17–20]. In a review, Glover and Hill [21] conclude that PNMS generally increases anxiety, depression, and stress responses in female offspring rather than in male; conversely, males are more likely to experience behavioral and learning problems following maternal insults. It has also been shown that prenatally stressed female mice have an increased vulnerability toward airway hyperresponsiveness and inflammation [22]. A growing body of evidence suggests that PNMS is associated with a range of sex-specific consequences in humans including altered head circumference-to-birth length ratios and poorer coordination abilities [23–25].

The process by which PNMS differentially affects girls and boys can be mediated by the placenta. In a review, Clifton [26] reports sex differences in placental cytokine expression, insulin-like growth factor pathways, and placental response to GC. Sood et al. [27] show that genes related to immune pathways are expressed differentially in female compared to male placenta. Stark et al. [28] report that high maternal GC concentrations lead to higher cortisol concentrations in cord blood and urine at 24 h after birth in females. These fetal-placental adjustments show that males and females adopt different coping strategies in the face of an adverse maternal environment [26]. Thus, a sex-based approach is warranted for research on PNMS.

To our knowledge, there are no published studies examining whether sex moderates the association between PNMS and asthma risk in humans. Yet, sex is recognized as an important factor for lung development and asthma [29]. Before puberty, asthma is more common in boys than girls (16.1% versus 10.5% in Canadian children aged 0–11 years [30]). In the postpubertal period, the sex ratio shifts towards females (9.8% in women, 7.4% in men [31, 32]). Underlying reasons for this shift remain unclear.

Past studies on the effect of prenatal maternal anxiety/stress have several limitations. While animal studies are capable of randomly assigning stressors to pregnant dams, their results are not directly applicable to humans [33]. Human research often studies life events, such as divorce and job loss, that may not be independent of the mother's characteristics. Finally, studies do not distinguish between the mother's objective exposure and her subjective reaction to a stressful event.

Natural disasters provide research opportunities with a number of methodological advantages. Disasters randomize the distribution of objective hardship, provide greater standardization of the nature and timing of the stressor, and allow the researcher to tease apart the effects of objective hardship and subjective distress.

In January 1998, 100 mm of freezing rain in the province of Quebec led to the most severe ice storm and one of the worst natural disasters, in Canada's history [34]. This event provided a unique opportunity to begin Project Ice Storm, a longitudinal prospective study of the effects of PNMS on child development. We have been following a cohort of women who were pregnant during the disaster and have conducted regular assessments of their children [24, 35–39]. As we became increasingly interested in immune functioning, an exploratory assessment was conducted to determine whether the stress experienced by pregnant women during the ice storm was associated with the risk of asthma in boys and/or girls. We conducted a phone survey to provide preliminary data until such time as more complete immune data from blood samples could be obtained. It was hypothesized that* in utero* exposure to higher levels of maternal objective hardship and/or subjective distress would be associated with increased rates of lifetime wheezing, doctor-diagnosed asthma, and inhaled corticosteroid usage in the children, particularly in girls.

## 2. Materials and Methods

### 2.1. Participants

Sixty-eight mothers from our Project Ice Storm cohort [39] accepted to complete a brief telephone interview concerning asthma symptoms in their children, their partners, and themselves, during the summer of 2011 when their children were 11 or 12 years of age. Data were collected on 32 boys and 36 girls and their parents.

### 2.2. Measures and Instruments

#### 2.2.1. Outcome Variables

*Asthma Outcomes.* A modified version of the International Study of Asthma and Allergies in Childhood (ISAAC) questionnaire was used to obtain information about asthma symptoms [40, 41]; questions about medical treatment and doctor-diagnosed asthma were added. Items were scored as having occurred or not. The present analyses focused on lifetime rates of wheezing, doctor-diagnosed asthma, and inhaled corticosteroid usage.

#### 2.2.2. Predictor Variables

*Objective Hardship.* In June 1998, the women's levels of objective hardship were assessed using a tailor-made questionnaire tapping into four categories of exposure: Threat, Loss, Scope, and Change [42]. Items were phrased to capture the objective facts of what happened to the women during the ice storm rather than how they felt about it.  Table 1 presents the questions that were used to assess the four dimensions. Each dimension was weighted equally because there was no theoretical reason to believe that any of the four dimensions had greater predictive value than the others [43]. A total objective hardship score, called Storm32, was calculated by summing scores from all four dimensions and is described in detail elsewhere [37]. In the Project Ice Storm cohort, this score has explained significant amounts of variance in several domains including obesity [35], insulin secretion [44], and cognitive and language development [38, 39] in childhood.

*Subjective Distress.* Subjective distress was assessed using a validated French version [45] of the Impact of Events Scale-Revised (IES-R) [46]. The IES-R is the most commonly used posttraumatic stress disorder (PTSD) screening instrument in disaster research. The scale describes symptoms from three categories relevant to PTSD: intrusive thoughts, hyperarousal, and avoidance. Scale items were written to reflect the mothers' enduring symptoms relative to the ice storm crisis. A score of 22 is often used as the threshold for a positive PTSD screening. The mean score for mothers in Project Ice Storm was 11.9 [47], compared to a mean score of 7.5 in a study of mothers 3 weeks postpartum [48] and a mean score of 4.8 for a control group of mothers 6 months postpartum [49]. Approximately 17% of the mothers had scores over 22, within the clinical range for potential PTSD [47]. High scores have been seen in war correspondents that had been shot at (mean IES-R = 20.2 [50]), health care workers in the SARS unit during the Toronto SARS outbreak (mean IES-R = 22.1 [51]), and Kosovar civilian war survivors (mean IES-R = 29.0 [52]). In Project Ice Storm the IES-R has explained variance in several domains including fetal growth [24] and dermatoglyphic asymmetry [36].

#### 2.2.3. Potential Confounding Variables

*Other Parental Characteristics.* Parental history of asthma was assessed by adding questions to the ISAAC questionnaire. The number of cigarettes the mother smoked per day during pregnancy was obtained from a postal questionnaire 6 months after the mothers' due date. Hollingshead socioeconomic status [53] was obtained in June 1998 and updated when the children were 11.5 years of age using a modified version of the Hollingshead scale. Lower scores indicate higher socioeconomic status.

Information concerning maternal life events was obtained when the children were 6 months and 11.5 years of age using a 29-item Life Experiences Survey (LES) [54]. Women indicated whether life events, such as a death or a promotion, had occurred in the preceding 18 months. The total number of different life events reported at 6 months and 11.5 years was used in the current analyses. We selected LES at 6 months (which covered the entire prenatal and early postpartum period) to be able to isolate the objective ice storm hardship from other potential stressors. Similarly, we included LES at the 11.5-year assessments to control for stressors that the mothers completing the survey may be experiencing and that could bias the results.

The 28-item General Health Questionnaire (GHQ-28) [55] was used to assess maternal psychological functioning in June 1998 and when the children were 11.5 years of age. The levels of GHQ depression and somatic symptoms were so low that there was no variance. Only the anxiety score was used in the present analyses. We included anxiety measures from June 1998 to isolate the anxiety assessed in the IES-R from generalized anxiety that the mother may have been experiencing. Moreover, we included a measure of anxiety at the 11.5-year assessment to control for the anxiety that mothers may have been experiencing at the time of the survey and which could potentially bias her responses to the medical questions. Obstetric complications were obtained using the Kinney Scale [56]. These included, but were not limited to, preeclampsia, use of forceps in delivery, and gestational diabetes. The McNeil-Sjöström Scale was used to rate the severity of all complications [57]. The total number of moderate-to-severe obstetric complications was used in the analyses.

*Other Child Characteristics.* Gestational age and birth weight were obtained from the children's “Vaccination Booklet,” completed by nursing staff after the birth. Timing of exposure to the ice storm was calculated based on the number of days between the peak of the storm (January 9, 1998) and the child's due date. A higher value indicates that the stress exposure occurred earlier during gestation.

### 2.3. Procedures

The Douglas Hospital Research Ethics Board approved all phases of Project Ice Storm. This pilot phase was approved on June 2012 (Protocol 10/23). Women pregnant during the ice storm were recruited in June 1998 by postal questionnaires. The first questionnaire, assessing the hardship and enduring reactions to the storm, the GHQ, and family demographics, was mailed to 1440 women by their obstetricians. A total of 224 women responded to this initial questionnaire, with 178 giving permission for further contact. Six months after each woman's due date, a second questionnaire assessing pregnancy outcomes was mailed. Of these, 177 women returned the second questionnaire. A number of subjects reported miscarriages or stillbirths, and many families were lost to follow-up during a gap in funding in the early years of the study. Since then, Project Ice Storm families have been assessed up to 17 times, including an assessment at age 11.5 years (*n* = 90). During the summer of 2010, 68 mothers who gave informed consent answered a phone interview on asthma when their children were either 11 or 12 years old.

### 2.4. Statistical Analyses

First, we conducted *t*-tests to compare the characteristics of the participating families versus the families who were in the original sample but did not answer the asthma questionnaire. We then compared families reporting asthma symptoms in their children according to demographic and potential risk variables using simple chi-squared and *t*-tests. Next, three hierarchical logistic regression models were conducted to predict three outcome variables: (1) the children's lifetime occurrence of wheezing or whistling in the chest, (2) doctor-diagnosed asthma, (3) and the children's lifetime inhaled corticosteroid utilization. A more liberal standard for rejection of the null hypothesis (i.e., *α* = 0.10) was adopted due to the exploratory nature of this pilot study and the small sample size [58]. The regression procedures included several blocks of variables; some blocks used Stepwise entry, while others forced predictor variables into the equation. Similar stepwise regression procedures have been used in other publications on the effects of PNMS [24, 35, 59]. The stepwise blocks used a *P* value of 0.10 to enter as a criterion. Potential confounding variables that were not significant were not included in the final model.

Child factors at birth (i.e., weight and gestational age) were allowed to enter into the equation during Block 1, in stepwise fashion. Parental factors (i.e., SES, obstetric complications, maternal anxiety (at 6 months and 11.5 years), maternal life events (6 months and 11.5 years), parental history of asthma, and number of cigarettes the mother smoked per day during pregnancy) were allowed to enter into the equation during Block 2, also by stepwise. The children's sex was forced into the equation during Block 3. Timing of the exposure to the stress was allowed to enter into the equation Block 4, in stepwise fashion. Objective hardship was forced into the equation during Block 5. Subjective distress was forced into the equation during Block 6. The interactions terms of objective hardship × sex of the child and subjective distress × sex of the child were entered in Block 7, in a stepwise fashion. Predictors that were not significantly related to the outcome measures were trimmed from the model due to the relatively small sample size and the analyses were reconducted. Hosmer-Lemeshow tests were conducted to provide information on the goodness of fit of the models.

## 3. Results

### 3.1. Descriptive Analyses

A comparison between the families who completed the asthma questionnaire versus families who responded to the June 1998 questionnaire but who did not answer the asthma questionnaire indicated that participating families had higher socioeconomic status levels (see Table 2). Moreover, participating mothers experienced a higher number of moderate-to-severe obstetric complications compared to nonparticipating mothers. Neither objective hardship, subjective distress nor other covariates, such as maternal anxiety, differed between responders and nonresponders.

### 3.2. Bivariate Associations

Descriptive statistics for the categorical variables (Table 3) and continuous variables (Table 4) are presented in relation to their associations with the three outcomes. Neither parental asthma status nor child sex was significantly associated with the outcomes. *t*-test results in Table 4 show that 3 of the 4 outcomes were significantly associated with a greater number of both perinatal (reported 6 months postpartum) and current life events. Although average SES was lower for children with symptoms, it was only significantly lower in the case of wheezing. Similarly, although there was a trend for more obstetric complications in children with symptoms, this was only significant in the case of corticosteroid use.

### 3.3. Hierarchical Logistic Regression

The final hierarchical logistic regression models for each outcome variable, after trimming of nonsignificant predictors, are presented in Table 5.

*Lifetime Wheezing or Whistling in the Chest.* Of the 68 children in the sample, 54.4% (20 boys and 17 girls) experienced wheezing or whistling in the chest. The initial model showed that neither objective exposure nor timing of exposure was significantly related to wheezing in boys or girls. The significant subjective stress-by-sex interaction indicated that the effect of* in utero* exposure to maternal subjective distress differed according to the sex of the child. Post-hoc analysis of the interaction showed that after controlling for current maternal life events, prenatal maternal subjective distress significantly predicted lifetime symptoms of wheezing or whistling in the chest in girls (OR = 1.11; 90% CI = 1.01–1.23); this association was not significant for boys. The Hosmer-Lemeshow test of goodness of fit indicated that the model adequately fit the data (*χ*_8_^2^ = 10.13, *P* = 0.26). The final model correctly classified 73.5% of cases, which is above the chance classification rate of 54.4%.

*Doctor-Diagnosed Asthma.* Mothers of 8 boys and 6 girls (20.6% of the sample) reported that a doctor diagnosed their child with asthma. Mothers who experienced more perinatal life events had children more at risk of being diagnosed with asthma by a doctor (OR = 1.27; 90% CI = 1.04–1.55). The effect of subjective distress on doctor-diagnosed asthma varied depending on the sex of the child (*P* = 0.02). Post-hoc analyses revealed that subjective distress was associated with a higher risk for doctor-diagnosed asthma in girls (OR = 1.09; 90% CI = 1.00–1.19), whereas it did not for boys (OR = 0.88; 90% CI = 0.78–0.99). The model adequately fit the data (*χ*_8_^2^ = 13.14, *P* = 0.11), and the model correctly classified 83.7% of cases, compared to the 79.4% classification rate by chance.

The available data also allowed us to analyze whether PNMS was associated with maternal-reported asthma. Six mothers reported that their child has asthma, although they did not report a doctor's diagnosis. In general, results for mother-reported asthma were similar to those for doctor-diagnosed asthma (results not shown).

*Lifetime Inhaled Corticosteroid Utilization. *There were 14 boys and 11 girls (36.8% of the sample) whose mothers reported that they had used inhaled corticosteroids. Results from the final model indicate that current maternal life events were associated with lifetime inhaled corticosteroids usage of their children (OR = 2.16; 90% CI = 1.34–3.48). Moreover, sex moderated the association between subjective distress and the children's lifetime utilization of inhaled corticosteroids (*P* = 0.03). Post-hoc analyses suggested that, after controlling for current maternal life events, the likelihood that a girl would have ever taken inhaled corticosteroids was associated with a 12% increase for every one point increase in subjective distress from the ice storm (OR = 1.12; 90% CI = 1.01–1.25). This effect was not seen for boys. The model adequately fit the data (*χ*_7_^2^ = 3.35, *P* = 0.85) and correctly classified 75.5% of cases, compared to classification by chance of 63%.

## 4. Discussion

Results of our pilot study are the first to suggest that disaster-related maternal subjective distress interacts with the child's sex to influence asthma risk. In girls only, higher levels of subjective maternal distress in pregnancy were associated with increased lifetime risk for wheezing, doctor-diagnosed asthma, and inhaled corticosteroid usage. Perinatal and current maternal life events were also associated with increased risk for asthma-related outcomes. Objective hardship and timing of the ice storm during pregnancy did not predict asthma-related outcomes in the final models with this sample, nor did they interact significantly with other predictors.

These results suggest that fetal immune function appears to have been influenced, in girls, by the mothers' posttraumatic stress-type symptoms from the ice storm rather than by what happened to them objectively, above and beyond the effects of other perinatal and current life events experienced by the mothers. Although further research is required, our findings support previous evidence suggesting that maternal distress during pregnancy opens a window for fetal programming of the immune system.

Although population rates of asthma during childhood naturally tend to be higher in boys than girls, results showed that PNMS was associated with the risk for asthma in girls only. Past studies have found that PNMS can masculinise some aspects of female development [60–65]. In a prospective study, Barrett et al. (2013) investigated the relationship between stressful life events during pregnancy and infant anogenital distance (AGD), a common sexually dimorphic biomarker of prenatal androgen exposure. Results showed that females who experienced higher levels of prenatal stress had significantly longer (i.e., more masculine) AGD than females who experienced less prenatal stress. Although our analyses and sample do not allow us to test this hypothesis directly, it would be interesting to examine whether PNMS increased rates of asthma in girls through a process of hormonal masculinisation.

A comprehensive understanding of the etiology of immune disorders in early childhood is necessary for effective primary prevention. In a study on developmental programming, Pincus and colleagues [66] propose that low levels of maternal progesterone, which can be triggered by activation of the HPA-axis, predict immune disorders, such as atopic dermatitis, in girls only. The authors also report that, in a murine model of stress-induced decrease in progesterone, supplements of a progesterone derivative abrogated the increase in allergic airway responsiveness observed in prenatally stressed offspring. Future human studies could monitor the effects of PNMS on progesterone levels and immune disorders in girls.

Our results also support the hypothesis that maternal life events during the perinatal and childhood periods are associated with an increased risk for asthma. Similarly, a twin study found that parental stress increased asthma morbidity in 1- and 3-year-old offspring [8]. Wright and colleagues [67] observed that caregiver stress is associated with increased total IgE expression and an enhanced allergen-specific proliferative response among 18–32-month olds. This highlights the need to consider both pre- and postnatal environments to better understand childhood asthma.

The present study has limitations. First, the observational design used in this study does not allow us to infer causation. Results only suggest that associations exist between PNMS exposure and asthma outcomes. Second, we did not have access to data on household pets or current parental smoking. Third, asthma symptoms may be under- or overreported by some mothers. Fourth, the sample size is small and thus can lead to an overestimation of the odds ratios. In addition, we did not have data on pubertal status of the subjects. Finally, replication of the study with a larger and more representative sample is required. The sample at baseline was not representative of the larger catchment area. Participants had higher SES scores than the regional averages and than nonresponders from the larger cohort. However, it is possible that the higher SES scores minimized the effects of PNMS rather than enhance it.

It is also worth noting that factors commonly related to wheezing and asthma in children were not found in the univariate analysis (e.g., parental asthma, gender). One explanation is that there may be different pathways by which asthma develops, as implied by the principle of equifinality, and that PNMS exposure weakens the genetic effects suggested by parental asthma; PNMS may be responsible for sporadic (nongenetic) cases of asthma. PNMS may also have altered the usual trends in sex differences, as noted above.

Despite the limitations, results of the present pilot study contribute to the PNMS literature. To our knowledge, this is the first prospective study of the effects of PNMS on asthma outcomes that assessed an independent, naturally randomized stressor. Moreover, the offspring's age in this sample increases the likelihood of accurate lifetime diagnoses for asthma compared to younger samples examined in other studies [13, 14].

We provided evidence that disaster-related PNMS may be associated with asthma outcomes in a sex-specific manner. Findings highlight the complexity of the association between PNMS and the fetal programming of chronic immune disorders. There is a need to produce evidence-based knowledge on the biological mechanisms by which a mother's subjective distress in the face of a natural disaster affects pre- and postnatal immune functioning over time. We suggest some directions for future research as follows.

*Future Research Directions*

- What roles do CG, sex hormones, and the placenta play in the development of immune disorders for prenatally stressed girls?
- Does PNMS cause fetal methylation of genes expressed in the lungs or lymphoid tissue that are involved in immune response function?
- Does PNMS induce asthma, does it exacerbate the disease, or does it speed up its progression?
- How do the biological mechanisms associated with objective hardship versus subjective distress differ?
- Do the effects of PNMS on immune disorders change over the lifespan?
- What interventions could prevent the effects of PNMS on immune disorders?

A multidisciplinary and longitudinal approach is necessary to complement our results and provide additional insights to prevent childhood immune disorders.

## Figures and Tables

**Table 1:** Questions used to assess the four dimensions (Threat, Loss, Scope, and Change) of objective prenatal maternal stress in the mothers after the ice storm.

Threat	Loss	Scope	Change
(1) Were you injured? No = 0 Yes = 1	(1) Did your residence suffer damage as a result of the ice storm? No = 0 Yes = 2	(1) How many days were you without electricity? 0 = 0–5 days 1 = 6–13 days 2 = 14–19 days 3 = 20-21 days 4 = >22 days	(1) Did your family stay together for the duration of the ice storm? Yes = 0 No = 1
(2) Was anyone close to you injured? No = 0 Yes = 1	(2) Did you experience a loss of personal income? No = 0 Yes = 2	(2) How many days were you without the use of your telephone? 0 = 0 days 1 = 0.01–1 day 2 = 2–4.5 days 3 = 5–7 days 4 = 8+ days	(2) Did you spend any time in a temporary shelter? No = 0 Yes = 1
(3) Were you ever in danger due to (3.1) …the cold No = 0 Yes = 1	(3) How much was the total financial loss including income, food, and damage to home? 0 = <$100 1 = $100–$1000 2 = $1000–$10000 3 = $10000–$100000 4 = >$100000		(3) How often were you required to change residence during the ice storm? 0 = 0 1 = 1 time 2 = 2+ times
(3.2) …exposure to downed electrical power lines No = 0 Yes = 1			(4) Did you take in guests during the ice storm? No = 0 Yes = 1
(3.3) …exposure to carbon monoxide No = 0 Yes = 1 (3.4) …lack of potable water No = 0 Yes = 1			(5) Did you experience an increase in physical work during the ice storm? 0 = less or same 1 = little or lot more
(3.5) …lack of food No = 0 Yes = 1			(6) Number of nights away from home: 0 = none 1 = 1–7.5 nights 2 = 8+ nights
(3.6) …falling branches and ice No = 0 Yes = 1			

8 points	8 points	8 points	8 points

**Table 2:** Comparison of characteristics between study responders and nonresponders.

Characteristics	Responders (*n* = 68)		Nonresponders (*n* = ~104)		*P* value
	Mean	SD	Mean	SD	
Anxiety	2.26	2.02	2.36	2.31	0.780
Total psychiatric symptoms	5.78	4.71	6.99	6.51	0.160
Subjective stress	11.02	12.29	12.01	12.82	0.620
Objective stress	11.71	3.79	10.88	4.52	0.200
Socioeconomic status	25.90	11.94	31.68	12.64	0.003

**Table 3:** Chi-squared tests providing descriptive statistics (*n*, %) for categorical variables.

Characteristics	Wheezing		Doctor-diagnosed asthma		Inhaled corticosteroids consumption	
	Yes (%) *n* = 37	No (%) *n* = 31	Yes (%) *n* = 14	No (%) *n* = 54	Yes (%) *n* = 25	No (%) *n* = 43
*Parent *						
Parental asthma						
Neither parent	23 (53)	20 (47)	8 (19)	35 (81)	16 (37)	27 (63)
At least one parent	14 (56)	11 (44)	6 (24)	19 (76)	9 (27)	16 (73)
Chi^2^ (1)	0.04, *P* = 0.84		0.28, *P* = 0.60		0.01, *P* = 0.92	
*Child *						
Sex						
Boy	20 (63)	12 (38)	8 (25)	24 (75)	14 (44)	18 (56)
Girl	17 (47)	19 (53)	6 (17)	30 (83)	11 (31)	25 (69)
Chi^2^ (1)	1.59, *P* = 0.21		0.72, *P* = 0.40		1.27, *P* = 0.26	

**Table 4:** *t*-tests providing descriptive statistics (Mean, SD) for continuous variables.

Variables	Wheezing in the chest			Doctor-diagnosed asthma			Inhaled corticosteroids consumption		
	Yes M (SD)	No M (SD)	*t*-test (df)	Yes M (SD)	No M (SD)	*t*-test (df)	Yes M (SD)	No M (SD)	*t*-test (df)
Socioeconomic status	**28.22** **(12.80)**	**23.13** **(10.34)**	−1.81* **(65.93)**	26.36 (10.70)	25.78 (12.33)	−0.16 (66)	26.16 (11.81)	25.74 (12.14)	−0.14 (66)
Maternal anxiety (6 months)	2.27 (1.94)	2.26 (2.14)	−0.03 (66)	2.71 (2.27)	2.15 (1.96)	−0.93 (66)	2.52 (2.06)	2.12 (2.00)	−0.79 (66)
Maternal anxiety (11.5 years)	1.13 (1.71)	0.69 (1.22)	−1.12 (53.83)	1.36 (1.96)	0.83 (1.39)	−1.06 (55)	1.15 (1.76)	0.81 (1.37)	−0.81 (55)
Life events (6 months)	**6.32** **(3.92)**	**4.61** **(2.74)**	−2.05** **(66)**	7.14 (4.29)	5.13 (3.20)	−1.64 (16.94)	**6.89** **(4.06)**	**4.77** **(2.94)**	−2.48** **(66)**
Life events (11.5 years)	2.19 (1.65)	1.46 (1.63)	−1.67 (56)	**2.58** **(1.83)**	**1.67** **(1.59)**	−1.71* **(56)**	**2.48** **(1.75)**	**1.51** **(1.54)**	−2.18** **(56)**
Obstetric Complications	5.00 (2.97)	4.26 (2.99)	−1.02 (66)	5.43 (2.79)	4.46 (3.02)	−1.08 (66)	**5.48** **(3.20)**	**4.19** **(2.77)**	−1.75* **(66)**
Child's birth weight (grams)	3345.95 (547.80)	3366.52 (601.24)	0.15 (66)	3313.87 (548.77)	3366.07 (578.05)	0.30 (66)	3362.77 (484.24)	3350.10 (617.69)	−0.08 (66)
Child's gestational age (weeks)	39.39 (2.01)	39.42 (1.90)	0.06 (66)	39.29 (2.19)	39.44 (1.90)	0.26 (66)	39.47 (1.76)	39.37 (2.07)	−0.20 (66)
Timing of stress (days)^§^	143.35 (89.45)	134.19 (76.00)	−0.45 (66)	168.21 (85.01)	131.65 (81.72)	−1.48 (66)	152.36 (92.14)	131.51 (77.48)	−1.00 (66)
Objective exposure	11.38 (4.32)	10.87 (4.25)	−0.49 (66)	12.00 (4.17)	10.93 (4.23)	−0.84 (66)	11.28 (4.62)	11.07 (4.10)	−0.20 (66)
Subjective distress	13.07 (13.64)	8.57 (10.12)	−1.52 (66)	13.00 (13.82)	10.50 (11.94)	−0.68 (66)	13.46 (14.97)	9.60 (10.35)	−1.25 (66)

*P < 0.10;  **P < 0.05; ^§^timing of stress is the number of days between the due date and January 9, 1998. A higher value indicates earlier exposure during gestation.

**Table 5:** Final logistic regression models predicting asthma-related outcomes.

Models	*B*	S.E.	Exp(*B*)	90% CI for Exp(*B*)	
**(1) Lifetime wheezing**					
Constant	−0.126	0.716	0.882		
Life events (11.5 years)	0.369*	0.227	1.447	0.997	2.100
Sex (0 = male)	−1.587*	0.918	0.204	0.045	0.926
Subjective distress	−0.030	0.038	0.971	0.912	1.033
Subjective distress × sex	0.137*	0.072	1.147	1.019	1.291

**(2) Doctor-diagnosed asthma**					
Constant	−1.655*	0.891	0.191		
Life events (6 months)	0.237*	0.121	1.267	1.039	1.545
Sex (0 = male)	−2.270*	1.181	0.103	0.015	0.721
Subjective distress	−0.126*	0.072	0.881	0.783	0.992
Subjective distress × sex	0.214**	0.088	1.238	1.071	1.431

**(3) Corticosteroid utilization**					
Constant	−0.999	0.823	0.368		
Life events (11.5 years)	0.769**	0.290	2.158	1.338	3.479
Parental asthma history	−1.661*	0.934	0.190	0.041	0.883
Sex (0 = male)	−1.759	1.103	0.172	0.028	1.058
Subjective distress	−0.057	0.047	0.945	0.874	1.021
Subjective distress × sex	0.173**	0.083	1.188	1.037	1.362

*P < 0.10; **P < 0.05.

Procedure: analyses were conducted on the entire sample (*n* = 69). Three distinct hierarchical logistic regression models were tested. Blocks of potential covariates used stepwise procedure with a *P* value of 0.10 as criterion to enter a variable into the model. Independent variables were forced into the model. Nonsignificant predictors were trimmed from the final model. Odds ratios (OR) and confidence intervals (CI) for interaction terms between stress measures and gender were calculated as per Kleinbaum and Klein [68].
